# Improvement of In Vitro Three‐Dimensional Cartilage Regeneration by a Novel Hydrostatic Pressure Bioreactor

**DOI:** 10.5966/sctm.2016-0118

**Published:** 2016-11-07

**Authors:** Jie Chen, Zhaoyuan Yuan, Yu Liu, Rui Zheng, Yao Dai, Ran Tao, Huitang Xia, Hairong Liu, Zhiyong Zhang, Wenjie Zhang, Wei Liu, Yilin Cao, Guangdong Zhou

**Affiliations:** ^1^ Department of Plastic and Reconstructive Surgery, Shanghai Key Laboratory of Tissue Engineering, Shanghai 9th People’s Hospital, Shanghai Jiao Tong University School of Medicine, Shanghai, People's Republic of China; ^2^ National Tissue Engineering Center of China, Shanghai, People's Republic of China; ^3^ Department of Anesthesiology, Shanghai 9th People’s Hospital, Shanghai Jiao Tong University School of Medicine, Shanghai, People's Republic of China; ^4^ Research Institute of Plastic Surgery, Wei Fang Medical College, Wei Fang, Shandong, People's Republic of China; ^5^ College of Materials Science and Engineering, Hunan University, Changsha, Hunan, People's Republic of China

**Keywords:** Tissue engineering, Hydrostatic pressure, Bioreactor, In vitro, Three‐dimensional cartilage regeneration

## Abstract

In vitro three‐dimensional (3D) cartilage regeneration is a promising strategy for repair of cartilage defects. However, inferior mechanical strength and tissue homogeneity greatly restricted its clinical translation. Simulation of mechanical stress through a bioreactor is an important approach for improving in vitro cartilage regeneration. The current study developed a hydrostatic pressure (HP) bioreactor based on a novel pressure‐transmitting mode achieved by slight deformation of a flexible membrane in a completely sealed stainless steel device. The newly developed bioreactor efficiently avoided the potential risks of previously reported pressure‐transmitting modes and simultaneously addressed a series of important issues, such as pressure scopes, culture chamber sizes, sealability, contamination control, and CO_2_ balance. The whole bioreactor system realized stable long‐term (8 weeks) culture under high HP (5–10 MPa) without the problems of medium leakage and contamination. Furthermore, the results of in vitro 3D tissue culture based on a cartilage regeneration model revealed that HP provided by the newly developed bioreactor efficiently promoted in vitro 3D cartilage formation by improving its mechanical strength, thickness, and homogeneity. Detailed analysis in cell proliferation, cartilage matrix production, and cross‐linking level of collagen macromolecules, as well as density and alignment of collagen fibers, further revealed the possible mechanisms that HP regulated in vitro cartilage regeneration. The current study provided a highly efficient and stable bioreactor system for improving in vitro 3D cartilage regeneration and thus will help to accelerate its clinical translation. Stem Cells Translational Medicine *2017;6:982–991*

**Significance Statement**

Inferior mechanical strength and tissue homogeneity of in vitro engineered three‐dimensional (3D) cartilage greatly restricted its clinical translation. The current study developed a hydrostatic pressure (HP) bioreactor based on a novel pressure‐transmitting mode, which efficiently avoided potential risks of the previously reported bioreactors in culture environment and contamination controls. The newly developed bioreactor realized stable long‐term culture under high HP and efficiently promoted in vitro 3D cartilage formation by improving its mechanical strength, cartilage regeneration thickness, tissue homogeneity, cell proliferation, extracellular matrix contents, and collagen cross‐linking level. This study provided a highly efficient and stable bioreactor system for improving in vitro 3D cartilage regeneration and thus helped to accelerate the clinical translation of this system.

## Introduction

Functional repair of cartilage defects is a challenging task because of the avascular and limited self‐repair nature of cartilage tissue [1, 2]. Tissue engineering is a promising approach to provide autologous engineered tissue for defect repair, and its feasibility in cartilage reconstruction has been widely demonstrated [3, 4]. Along with technology development and ascending requirements of clinical translation, in vitro tissue regeneration gradually becomes an important research direction because of such advantages as low cell loss, low inflammatory reaction, ease of handling, standard manufacture, and controllable quality [5, 6]. As a result of its avascular nature, cartilage is one of the earliest tissues to have been successfully engineered in vitro. However, tissue structure and function of the in vitro engineered cartilage (vitro‐EC) are still inferior to those of the native cartilage [1]; this has become one of the main obstacles impeding clinical application.

Compared with native cartilage, major deficiencies of vitro*‐*EC lie in its poor mechanical properties, inferior homogeneity, and low amount of cartilage‐specific extracellular matrices (ECMs) [7, 8]. These deficiencies are mainly caused by the differences between the in vitro culture condition of the engineered cartilage and the developmental microenvironment of the native one [9]. To address these deficiencies, in vitro simulation of the physiological microenvironment has been generally accepted as a feasible approach. Mechanical loading is considered one of the most important features of the articular physiological environment that regulates the development and maturation of articular cartilage [10]. During joint movement, hydrostatic pressure (HP) from synovial fluid is the major mechanical stress [11]. Therefore, many studies tried to simulate this kind of mechanical stress by means of bioreactors and demonstrated their roles in promoting in vitro cartilage formation and enhancing biomechanical properties and ECM deposition of vitro‐EC [12, 13].

The key point of simulating HP is how to transmit the pressure to the culture medium. The reported HP bioreactors mainly transmitted the pressure through fluid phase by means of a piston or direct gas phase [14, 15, 16]. For the fluid phase mode, the piston directly contacted the culture medium while transmitting the pressure by piston movements, which apparently had a very high requirement for sealability and contamination control [14, 15]. For direct gas phase mode, a pressurizing gas phase would alter the gas concentration within the culture medium and thus might have an uncontrolled effect on the cultured tissue [16]. Therefore, it is very necessary to design a novel pressurizing mode that may not only achieve an ideal control in sealability and contamination but also avoid the influences of gas pressure‐transmitting modes on the culture environment.

To address these issue, the current study designed and developed a novel HP bioreactor that could transmit the pressure to the culture medium through a flexible membrane in a completely sealed stainless steel chamber. This pressurizing mode might lower the risk for contamination caused by the piston movements and avoid the potential influences of gas pressure change on the cultured tissue, thereby simultaneously avoiding the problems of a piston‐based fluid phase pressure bioreactor and a direct gas phase pressure bioreactor. In addition, the culture chamber of the bioreactor was designed to have a cavity large enough for in vitro culture of multiple samples or large three‐dimensional (3D) tissue.

The current study demonstrated the feasibility of this novel bioreactor for long‐term in vitro culture of 3D cartilage generated by a tissue engineering model. Moreover, the efficacy, advantages, and underlying mechanisms of HP provided by the newly developed bioreactor were evaluated in tissue, cell, and molecular levels by comparison with two other types of reported mechanical stimuli (shear stress and centrifugal force with the reported optimized parameters [17, 18]) and static culture using the same 3D cartilage engineering model.

## Materials and Methods

### Design and Development of Novel HP Bioreactor

**Figure 1:**
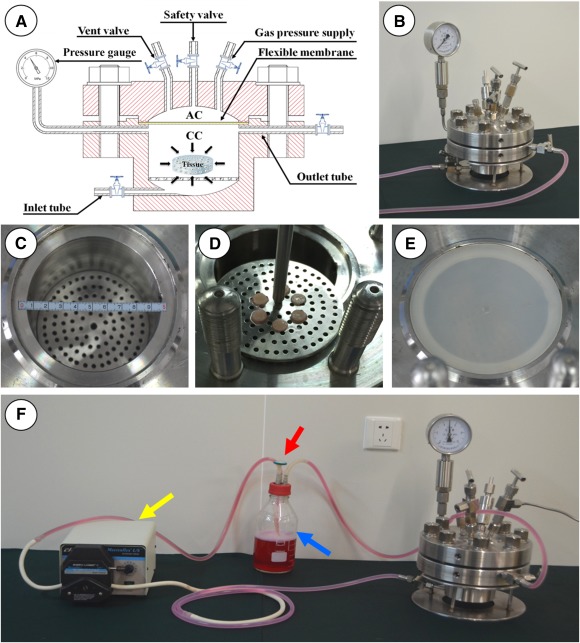
Self‐designed hydrostatic pressure (HP) bioreactor. **(A):** Schematic representation of HP bioreactor. **(B):** Main device of HP bioreactor system. **(C):** Internal structure of culture chamber (CC). **(D):** Tissue sample stage of CC. **(E):** Flexible membrane on CC. **(F):** HP bioreactor system. In the pressurizing mode, both inlet and outlet valves are closed. The high‐pressure nitrogen gas enters into the air chamber through the gas pressure supply channel and transmits the pressure to the flexible membrane, which transmits the pressure into the culture medium in the completely sealed CC. The pressure gauge provides a real‐time pressure reading. During nonpressuring mode, both inlet and outlet valves are opened. The culture medium is circulated continuously between the storage bottle (blue arrow) and the CC is driven by the peristaltic pump (yellow arrow), which guarantees the CO_2_ balance of the medium by means of a filter (red arrow) in the storage bottle.

The novel HP bioreactor was designed and fabricated by our group and Hu Nan University. The HP bioreactor system (Fig. 1) consisted of a stainless steel device, a high‐pressure nitrogen gas receptacle, a peristaltic pump, and a culture medium storage bottle. The stainless steel device (Fig. 1A, 1B) was composed of a culture chamber (Fig. 1C) with a moveable tissue sample stage (Fig. 1D), an air chamber, a flexible membrane (Fig. 1E), a pressure gauge, and several valves.

Detailed design and working principle are shown in Figure 1. The whole bioreactor system (except for the nitrogen gas and the peristaltic pump) was placed inside an incubator with 37°C, 95% humidity, and 5% CO_2_.

### Isolation and Culture of Articular Chondrocytes

Three newborn swine were purchased from Shanghai Jia Gan Experimental Animal Raising Farm (Shanghai, China) and used for triple‐repeat experiments. All animal study protocols were approved by the Animal Care and Experiment Committee of Shanghai Jiao Tong University School of Medicine. Chondrocytes were harvested from the surface cartilage of knee joints according to the previous method established in our laboratory [19].The harvested cells were then seeded into 10‐cm culture dishes at a final concentration of 1.5 × 10^4^ per cm^2^ in DMEM (Hyclone, GE Healthcare Life Sciences, Pittsburgh, PA, http://www.gelifesciences.com) with 10% fetal bovine serum (Hyclone) and 1% penicillin‐streptomycin (regular medium). Cells in passage 2 were used for in vitro cartilage construction.

### Preparation and In Vitro Culture of Cell‐Scaffold Constructs

Fifteen microgram of unwoven polyglycolic acid (PGA) fibers (synthesized by National Tissue Engineering Center of China, Shanghai, People's Republic of China, http://en.sjtu.edu.cn/research/centers‐labs/national‐tissue‐engineering‐research‐center) was compressed by a silicone rubber mold into a disk 9 mm in diameter and 2.5 mm in thickness. We regularly added 0.3% polylactic acid (PLA) (Sigma‐Aldrich, St. Louis, MO, http://www.sigmaaldrich.com), diluted in dichloromethane solvent, was added repeatedly to the PGA disks until the PLA content reached approximately 10% of the PGA/PLA scaffold mass [18, 20].

The scaffolds were disinfected with 75% ethanol solution for 40 minutes, followed by double washes with phosphate‐buffered saline (PBS). Chondrocytes in passage 2 were harvested and seeded into the PGA/PLA scaffolds at a concentration of 8.0 × 10^7^ cells/ml (80 μl in each scaffold), followed by a 4‐hour incubation as previously reported, and then cultured in regular medium [18, 20]. All the constructs were cultured in six‐well plates at 37°C, 95% humidity, and 5% CO_2_ for 4 weeks before grouping study.

### Mechanical Stimulation Modes

After 4 weeks of in vitro culture, all the constructs were divided randomly into four groups according to the culture modes. In the HP group, the constructs were cultured in the current HP bioreactor, and an extra HP stimulation at 5 MPa for 30 minutes twice a day was added for another 8 weeks [21, 22]. In the shear stress control group (shear group), the constructs were cultured in a spinner flask with a continuous spinning speed of 30 rpm for another 8 weeks, as described previously [17]. In the centrifugal force control group, the constructs were cultured in 50‐ml centrifugal tubes, and an extra centrifugal force stimulation at 100*g* for 30 minutes twice a day was added for another 8 weeks [18]. In the static culture control group (static group), the constructs were cultured in six‐well plates at 37°C, 95% humidity, and 5% CO_2_ with no mechanical stimulations for another 8 weeks.

### Wet Weight and Thickness

All vitro‐EC samples in the different groups were weighed with an electronic balance, and their thicknesses were measured with a vernier caliper. The differences in wet weight and thickness among groups were analyzed by one‐way analysis of variance.

### Histological and Immunohistochemical Evaluations

After 12 weeks of in vitro culture, the vitro‐EC samples were fixed in 4% paraformaldehyde, embedded in paraffin, and sectioned into 5‐μm slices. Sections were stained with hematoxylin and eosin and Safranin‐O to evaluate tissue structure and cartilage ECM deposition. For immunohistochemical analysis, expression of type II collagen was detected by mouse anti‐human type II collagen monoclonal antibody (1:100 in PBS; Santa Cruz Biotechnology, Santa Cruz, CA, https://www.scbt.com/), followed by horseradish peroxidase‐conjugated anti‐mouse antibody (1:200 in PBS; Santa Cruz Biotechnology) and color development with diaminobenzidine tetrahydrochloride (Santa Cruz Biotechnology) as described previously [20].

### Glycosaminoglycan Quantification

Glycosaminoglycan (GAG) content of vitro‐EC in different groups (*n* = 6 in each group) was quantified by dimethyl methylene blue chloride (Sigma‐Aldrich) [23]. Total GAG was precipitated by guanidinium chloride solution (0.98 mol/L). After the GAG precipitate was dissolved, the optical density (OD) was determined at 595 nm. A standard curve was established according to the OD values of chondroitin‐4‐sulfate with different concentrations. The total GAG amounts were determined on the basis of the OD value and the standard curve.

### Total Collagen Quantification

The content of total collagen in different groups (*n* = 6 in each group) was quantified by a hydroxyproline assay. The samples were prepared by alkaline hydrolysis, and free hydroxyproline hydrolysates were assayed according to previously described methods [24]. The hydroxyproline content was finally converted to total collagen according to the mass ratio of collagen to hydroxyproline of 7.25.

### DNA Quantification

The fluorescent PicoGreen double‐stranded DNA quantification assay (Thermo Fisher Scientific Life Sciences, Waltham, MA, http://www.thermofisher.com) was used to analyze DNA contents of vitro‐EC in different groups (*n* = 6 in each group). Samples were treated and examined according to a previously reported method [25]. The working reagent solution was prepared as a 200‐fold dilution of the concentrated dimethyl sulfoxide solution in 1× TE (20 mM Tris‐HCl; 2 mM EDTA; pH 7.5). Undiluted sample digest (100 µl) was mixed in a 1:1 ratio with the working solution and incubated for 5 minutes at room temperature (with light avoided) and then excited at 480 nm. Fluorescence emission intensity was measured at 520 nm and compared against a standard curve.

### Biomechanical Examinations

Young’s modulus detected by a biomechanical analyzer (Instron‐5542, Instron, Canton, MA, http://www.instron.us) was used for biomechanical tests in a manner similar to previously established methods [20]. All the samples in different groups (*n* = 6 in each group) were applied with a constant compressive strain rate of 0.5 mm/minute until 80% of maximal deformation was achieved. The Young’s modulus of the tested samples was calculated on the basis of the slope of the stress‐strain curve.

### Enzyme‐Linked Immunosorbent Assay

The samples in different groups (*n* = 6 in each group) were completely homogenized to release ECM proteins, and the supernatant was obtained for enzyme‐linked immunosorbent assay (ELISA) according to a previously reported method [26]. Lysyl oxidase (LOX) and pyridinoline (PYR) were quantified by the swine LOX and PYR ELISA kits (R&D Systems, Minneapolis, MN, http://www.rndsystems.com).

### Quantitative Real‐Time Reverse Transcription‐Polymerase Chain Reaction

RNA was extracted from the samples in different groups (*n* = 6 in each group) and reversely transcribed into cDNA as previously reported [27]. Polymerase chain reaction (PCR) was conducted by using a SYBR green reaction Kit (Roche Molecular Biochemicals, Mannheim, Germany, https://molecular.roche.com) and the corresponding gene primers. The expression genes were analyzed by using an ABI 7300 Real‐time PCR System (Thermo Fisher). The expression of *LOX* gene was normalized by comparison with the housekeeping gene *β‐actin*. The primer sequences for *LOX* and *β‐actin* are listed in supplemental online Table 1.

### Transmission Electron Microscope

According to a previous method [28], the samples in different groups were cut into 1.0‐mm^3^ sections, rinsed with PBS, fixed in 2.5% glutaraldehyde at 4°C overnight, and then embedded in epoxy resin. The samples were sectioned into 20‐30 nm slices for transmission electron microscopy (TEM) analysis.

### Statistical Analysis

All the quantitative data were recorded as mean ± SD. After confirmation of a normal data distribution, one‐way analysis of variance and post hoc least‐significant‐difference tests were used to determine statistical significance among groups (GraphPad Prism version 6.0; GraphPad Software, La Jolla, CA, http://www.graphpad.com). A *p* value <.05 was considered to indicate a statistically significant difference.

## Results

### Construction of Cartilage Regeneration Model In Vitro

After PLA coating, unwoven PGA fibers (supplemental online Fig. 1A) could be easily prepared onto a cylindrical scaffold with a diameter of 9 mm and a thickness of 2.5 mm (supplemental online Fig. 1B). Scanning electron microscopy (SEM) revealed that the scaffold presented a porous structure (supplemental online Fig. 1C). Chondrocytes in passage 2 (supplemental online Fig.1D) could easily adhere to the PGA fibers to form a cell‐scaffold construct (supplemental online Fig. 1E). After 3 days of in vitro culture, SEM examination demonstrated that chondrocytes had attached to the scaffold and secreted ECM to cover the PGA fibers (supplemental online Fig. 1F).

### Effect of HP on Macroscopy and Histology of Vitro‐EC

**Figure 2:**
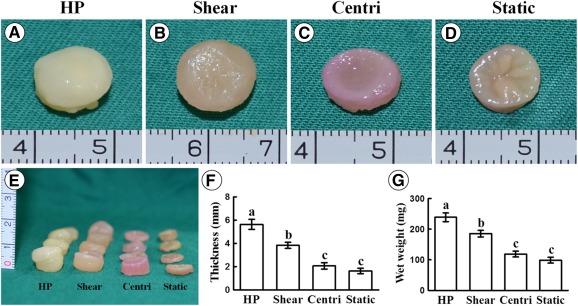
Gross view, thickness, and wet weight of in vitro engineered cartilage. **(A–E):** Samples in different groups show different color and texture in appearance with visible differences in sample thickness. Quantitative analyses show that both thickness **(F)** and wet weight **(G)** are significantly different (*p* < .05) among groups, except for the wet weight between the centri and static groups. Error bars indicate SD; a–c, *p* < .05. Abbreviations: centri, centrifugal force control group; HP, hydrostatic pressure.

Whether HP provided by the current bioreactor had significant effects on in vitro cartilage formation was an important issue in the current study. According to the current results, after 12 weeks of in vitro culture with or without different mechanical stimulations, all the samples in different groups formed cartilage‐like tissue and basically maintained the original shape. However, samples in different groups showed different color and texture in appearance, with significant differences in thickness and wet weight. The samples in the HP group showed a smooth, compact, ivory‐white appearance and achieved the maximal wet weight and thickness among groups, indicating the optimal cartilage formation. The samples in the shear group also showed a relatively smooth surface with faint yellow appearance and achieved higher thickness and wet weight compared with the centrifugal force and static groups. The samples in both the centrifugal force and static groups showed irregular surfaces with obvious pits in the central area, and no significant differences were observed in thickness or wet weight between these two groups (Fig. 2).

**Figure 3:**
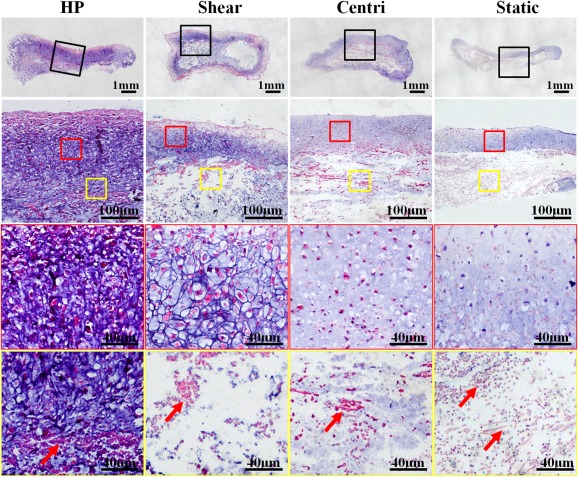
Hematoxylin and eosin staining of in vitro engineered cartilage. The sample in the HP group shows relatively homogeneous cartilaginous features with abundant lacuna structures, strong extracellular matrix staining, and the thickest cartilage formation. Samples in the shear and centri groups also form cartilaginous tissue with a certain thickness, but obvious heterogeneous structures are observed in the central regions. Cartilage formation in the static group is observed only in the outer region of the sample. Red frames indicate the outer cartilaginous regions and yellow frames indicate the central regions. Red arrows indicate polyglycolic acid residuals. Abbreviations: centri, centrifugal force control group; HP, hydrostatic pressure.

Histological examinations further supported the macroscopy results. Generally, all the samples with mechanical stimuli (HP, shear, and centrifugal force groups) showed more compact tissue structure and cartilage‐specific ECM depositions compared with the samples without mechanical stimuli (static group). Among the groups with mechanical stimuli, the HP group achieved the thickest cartilage formation with relatively homogeneous tissue structures, abundant lacuna, and strong cartilage‐specific ECM staining. In the shear and centrifugal force groups, the samples also formed cartilaginous tissue with a certain thickness, but these samples presented obvious heterogeneous structures, in which cartilage‐specific ECM depositions were observed only at the outer regions but not the central regions. In the static group, only a thin layer of cartilage‐like tissue with weak cartilage‐specific ECM staining was observed at the outer region (Fig. 3; supplemental online Figs. 2, 3). Noticeably, the central regions of all the samples in the shear, centrifugal force, and static groups but not in HP group showed loosened structures with obvious residue of undegraded PGA fibers (Fig. 3; supplemental online Figs. 2, 3). In addition, positive expressions of type I collagen at different extents were observed in the shear, centrifugal force, and static groups but not in HP group, indicating that HP might help to downregulate expression of type I collagen because of better simulation of articular mechanical environment (supplemental online Fig. 4). All these results indicated that the HP provided by the current bioreactor played a more important role in promoting in vitro cartilage formation under the current 3D cartilage regeneration model compared with all the control groups.

### Effect of HP on ECM Contents of vitro‐EC

**Figure 4:**
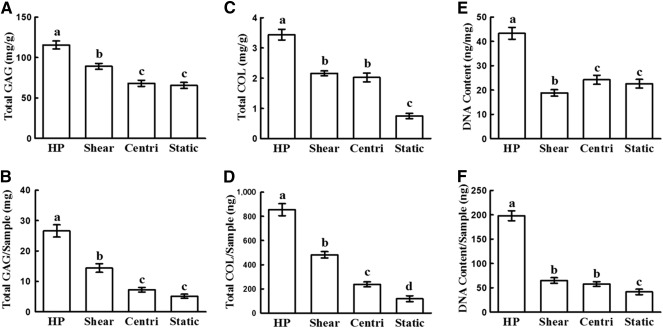
Quantitative evaluation of cartilage formation. **(A–E):** The quantitative analyses show that the HP group achieved the highest contents of total GAG, total COL, and DNA, with significant differences (*p* < .05) among groups. **(A–D):** The shear group showed greater content of total COL and total GAG, with significant differences (*p* < .05) compared with those in static group. **(C, D):** In the centri group, only total COL contents showed significant differences (*p* < .05) compared with the static group. Error bars indicate SD; a–d, *p* < .05. Abbreviations: centri, centrifugal force control group; COL, collagen; GAG, glycosaminoglycan; HP, hydrostatic pressure.

Quantitative analysis of ECM contents revealed a consistent trend in sample thickness and wet weight, which further confirmed the results of macroscopic and histological examinations. As shown in Figure 4, the HP group achieved the highest contents of total GAG and total collagen among groups with significant differences (*p* < .05). The shear group also showed higher contents of total collagen and total GAG compared with those in the centrifugal force and static groups, although much lower than those in the HP group. For the centrifugal force group, only total collagen content was a little higher than that in the static group. Noticeably, only the HP group achieved a remarkable increase in DNA contents, with significant differences compared with all the control groups. These results demonstrated that HP provided by the current bioreactor not only significantly enhanced ECM production of vitro‐EC but also played an important role in promoting cell proliferation.

### Effect of HP on Mechanical Properties and Collagen Cross‐Linking of Vitro‐EC

**Figure 5:**
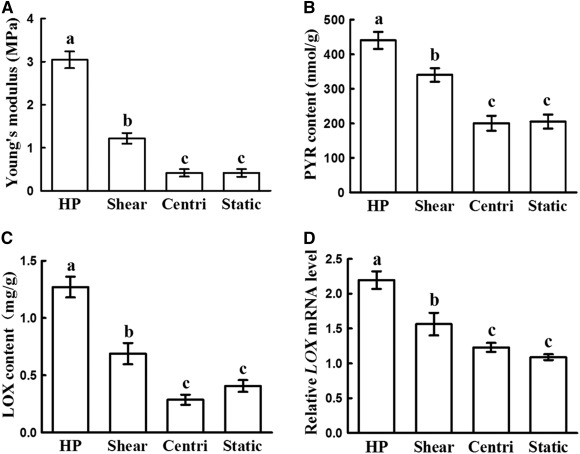
Young’s moduli and expressions of LOX and PYR. Young’s modulus **(A)**, PYR content **(B)**, LOX content **(C)**, and *LOX* gene expression **(D)** in the HP and shear groups were significantly higher than those in the centri and static groups (*p* < .05). All these quantitative evaluations in the HP group were significantly higher than those in shear group (*p* < .05). No significant differences were observed between the centri and static groups. Error bars indicate SD; a–c, *p* < .05. Abbreviations: centri, centrifugal force control group; HP, hydrostatic pressure.

**Figure 6:**
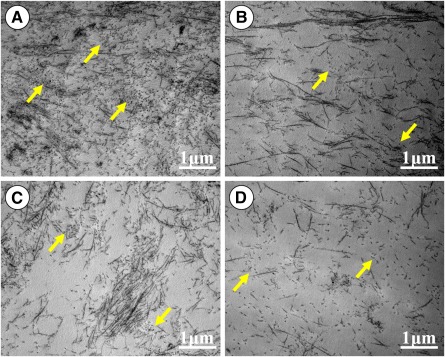
Transmission electron microscopy examinations of in vitro engineered cartilage. The density of collagen fibers decreased in sequence among groups in order of hydrostatic pressure (HP) **(A)**, shear **(B)**, centrifugal force **(C)**, and static **(D)**. Relatively uniform distributions of collagen fibers were observed in the HP **(A)** and shear **(B)** groups but not in the centrifugal force **(C)** and static **(D)** groups. Yellow arrows indicate perpendicularly distributed collagen fibers.

Whether HP provided by the current bioreactor helped to improve mechanical properties of vitro‐EC was the issue of most concern in the current study. As shown in Figure 5, the HP group achieved the highest Young’s modulus among groups with significant differences (*p* < .05) (Fig. 5A). The shear group also showed a higher Young’s modulus compared with those in centrifugal force and static groups, although a much lower one than the HP group (Fig. 5A). No significant differences were observed in Young’s modulus between the centrifugal force and static groups (Fig. 5A). These results were highly consistent with the collagen cross‐linking level, where HP group achieved the highest PYR and LOX contents as well as relative LOX mRNA level (Fig. 5B–5D), implying that HP provided by the current bioreactor might help to promote collagen cross‐linking and thus enhance the mechanical properties of vitro‐EC. TEM examinations also revealed that high‐density collagen fibers with relatively uniform distributions were observed only in the HP groups but not in the shear, centrifugal force, and static groups (Fig. 6), which further supported the results of mechanical analysis.

## Discussion

Inferior mechanical strength of in vitro engineered 3D cartilage is one of the major obstacles that restricted its clinical translation. Many studies reported the significant roles of HP on in vitro cartilage regeneration and developed many kinds of HP bioreactors based on different pressure‐transmitting modes [14, 15, 16, 29, 30]. However, the previously reported HP bioreactors were not suitable for long‐term culture in vitro of a large cartilage in terms of pressure‐transmitting modes, pressure scopes, culture chamber sizes, and contamination control, and other factors [14, 15, 16]. Moreover, it is still unclear how HP regulates in vitro 3D cartilage regeneration and improves its mechanical properties. The current study developed an HP bioreactor based on a novel pressure‐transmitting modes, which efficiently addressed the above issues in the same system and realized long‐term culture under high HP without medium leakage and contamination. Furthermore, in vitro 3D tissue culture revealed that the current bioreactor efficiently promoted in vitro 3D cartilage regeneration by improving its mechanical strength, engineered cartilage thickness, and tissue homogeneity. Detailed analysis of cell proliferation, cartilage matrix production, cross‐linking level of collagen macromolecules, and density and alignment of collagen fibers further revealed the possible mechanisms through which HP regulated in vitro cartilage formation. These results indicated that the newly developed bioreactor was an efficient and stable system for improving in vitro 3D cartilage regeneration.

An ideal HP bioreactor for in vitro cartilage engineering should include several key characteristics: (a) a proper pressure‐transmitting mode that does not change the culture environment; (b) good sealability for contamination control; (c) tunable pressure control with a maximal pressure close to physiological scope (3–10 MPa) [31]; (d) a chamber large enough for multiple samples or large sample; and (e) easy control in culture medium exchange and CO_2_ balance. Few of the previously reported HP bioreactors achieved all the above requirements simultaneously. For example, the direct gas phase pressurizing mode inevitably increased the gas pressure in medium and thus altered the culture environment [16], whereas the piston‐based fluid phase pressurizing mode probably led to difficulties in controlling sealability and contamination.

In contrast to these pressurizing modes [14, 15, 16], the current HP bioreactor introduced a novel pressurizing mode that transmitted the pressure to the culture medium through the slight deformation of a flexible membrane. This mode was able to achieve a complete seal and thus prevented possible contamination. Importantly, the pressurizing gas was separated from the medium by the flexible membrane, which completely avoided the influence of high‐pressure gas on the culture environment during the pressurizing period. In addition, the culture chamber was designed to be large (750 ml in volume, 9 cm in diameter) and was fabricated by stainless steel with a thickness of 1.5 cm; thus, it was solid enough to withstand the high HP and was suitable for long‐term culture of large tissue. In addition, the whole bioreactor could be placed into the CO_2_ incubator and the culture medium could circulate continuously between the storage bottle and culture chamber under the drive of the peristaltic pump, which guaranteed the CO_2_ balance of the medium by means of a filter in the storage bottle. Altogether, all these characteristics made the current HP bioreactor suitable for long‐term culture of 3D vitro‐EC.

Despite this design, whether this bioreactor could work stably as designed and efficiently improve in vitro cartilage formation was still an important issue. Our pilot study had preliminarily demonstrated the long‐term stability of this bioreactor system for in vitro culture before it was used for formal tissue culture (data not shown). Furthermore, all the triple repeated experiments for in vitro 3D cartilage engineering also demonstrated that the bioreactor system could stably run for 8 weeks without medium leakage and contamination. Most important, the results of 3D cartilage regeneration in vitro revealed that the HP group achieved the optimal cartilage formation compared with the other groups, with significant differences in cell proliferation, wet weight, cartilage thickness, tissue homogeneity, cartilage ECM contents, and mechanical properties. These findings indicate that HP provided by the current bioreactor might be a relatively more appropriate mechanical stimulation for promoting in vitro cartilage regeneration, at least for the current model of cartilage construction.

After the efficiency of this novel bioreactor was shown, how HP promoted in vitro cartilage formation became another important issue. According to the current results, the main mechanism of HP might include the following. The first is the promotion of cell proliferation and ECM production, which was consistent with other reports. Previous studies reported that HP could regulate some key signal pathways [32, 33, 34, 35, 36], stimulate secretion of growth factors [37, 38], and thus promote cell proliferation and ECM production, which was also confirmed by the current results.

A second mechanism could be improving nutrient and metabolite transportation, which was a new finding in the current study. It is accepted that in vitro 3D cartilage regeneration has a thickness limit due to the difficulty with nutrient and metabolite transportation [39]. During in vitro 3D cartilage regeneration, cartilage formation at the outer layer might block the substance exchange of the inner part to interfere with cartilage formation and thus result in a "hollow" in the central region [40]. It is speculated that HP can promote substance exchange by increasing diffusion pressure and thus helps to improve cartilage formation in the inner part, which may be an important reason that the cartilage thickness in the HP group was significantly higher than that in the other groups.

A third mechanism is enhanced cross‐linking of cartilage ECM macromolecules, which was another important finding in the current study. Cross‐linking level of the ECM macromolecules is the important factor that determines the mechanical properties of vitro‐EC [41]. According to the current results, PYR (the main cross‐linking molecule) content, LOX (the key enzyme of regulating cross‐linking) level, and Young’s modulus in the HP group were significantly higher than those in the other groups, indicating that HP might improve mechanical properties of vitro‐EC by enhancing the cross‐linking level of the ECM macromolecules. This was further supported by density and alignment of collagen fibers on SEM examinations. All these findings provide new explanations for the roles and mechanisms of HP in regulating in vitro 3D cartilage regeneration.

Although the current results demonstrated the important roles and possible mechanism of the novel HP bioreactor in promoting in vitro cartilage formation, it is difficult to evaluate whether the current system is superior to the other reported systems because the different research models for scaffold types, cell sources, HP‐related parameters, culture medium components, and in vitro culture time might greatly influence cartilage regeneration. For the current system, some detailed parameters (such as stimulation amplitude, frequency, start time, and duration) and exact mechanisms still need to be further investigated. In addition, the current bioreactor only simulated the mechanical loading of the articular environment in a resting state. To further simulate the physiological stimuli during joint movements, it is necessary to modify the current bioreactor to provide dynamic HP for in vitro cartilage formation. In addition, chondrogenic growth factors (such as transforming growth factor‐β1 and insulin‐like growth factor‐1) [42, 43] should also be introduced in the culture system to further improve in vitro cartilage formation because the roles of these factors in enhancing cartilage regeneration have been widely testified. All these proposed strategies should be investigated in future studies.

## Conclusion

The current study developed an HP bioreactor based on a novel pressure‐transmitting mode, which efficiently avoided the potential risks of previously reported pressure‐transmitting modes in culture environment and contamination controls. The current bioreactor realized stable long‐term culture under high HP without medium leakage and contamination, and it efficiently promoted in vitro 3D cartilage regeneration by improving its mechanical strength and tissue homogeneity. Moreover, the underlying mechanisms that HP regulated in vitro cartilage formation were also explored in tissue, cell, and molecular levels. Although more detailed parameters, exact mechanisms, and some new strategies should be further investigated, the current study demonstrated an efficient and stable bioreactor system for improving in vitro 3D cartilage regeneration and thus may help accelerate the clinical translation of this system.

## Author Contributions

J.C.: conception and design, provision of study material or patients, collection and/or assembly of data, data analysis and interpretation, manuscript writing; Z.Y.: provision of study material or patients, collection and/or assembly of data, data analysis and interpretation, manuscript writing; Y.L.: data analysis and interpretation, manuscript writing; R.Z. and R.T.: provision of study material or patients, collection and/or assembly of data; Y.D. and H.L.: conception and design, provision of study material or patients; H.X.: collection and/or assembly of data, data analysis and interpretation; Z.Z., W.Z., and W.L.: administrative support, final approval of manuscript; Y.C.: conception and design, final approval of manuscript, financial support; G.Z.: conception and design, administrative support, manuscript writing, final approval of manuscript, financial support.

## Disclosure of Potential Conflicts of Interest

The authors indicated no potential conflicts of interest.

## Supporting information

Supporting Information
