# Supplementary material for: Improvement of In Vitro Three‐Dimensional Cartilage Regeneration by a Novel Hydrostatic Pressure Bioreactor
Source: Stem Cells Transl Med. 2016 Nov 7;6(3):982–91. doi: 10.5966/sctm.2016-0118 (PMC5442788; doi:10.5966/sctm.2016-0118)
Supplement: Supplementary file 1 — Supporting Information [file SCT3-6-0982-s001.pdf]

Supplemental Figures – Zhou et al.

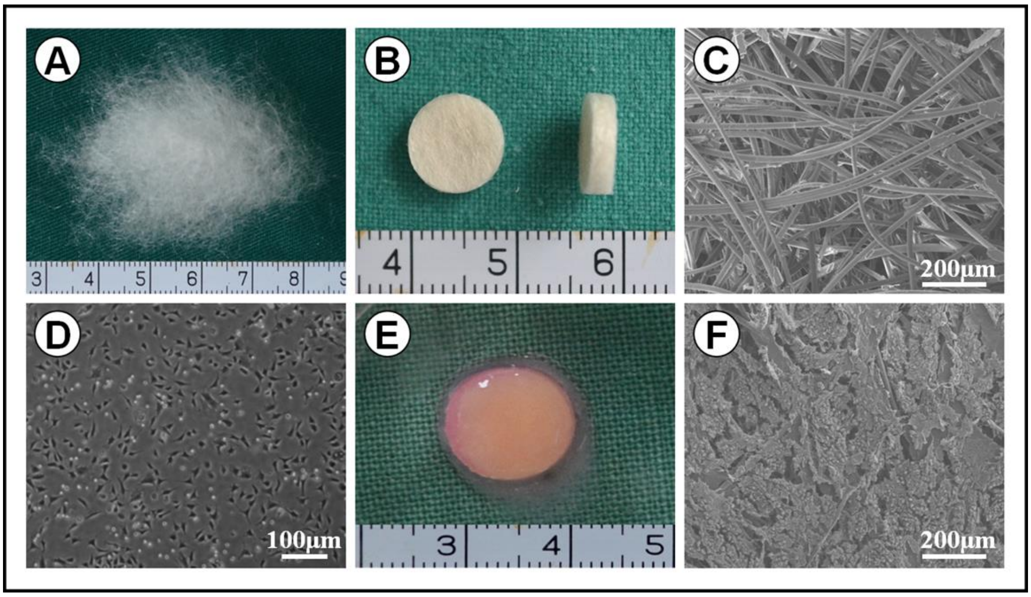

Figure S1: Preparation and characterization of the cell-scaffold constructs. A: Unwoven PGA fibers; B: PGA/PLA scaffold; C: SEM of PGA/PLA scaffold; D: Chondrocytes in passage 2; E: Chondrocyte-PGA/PLA construct; F: SEM of chondrocyte-PGA/PLA construct after 3 days of cell seeding.

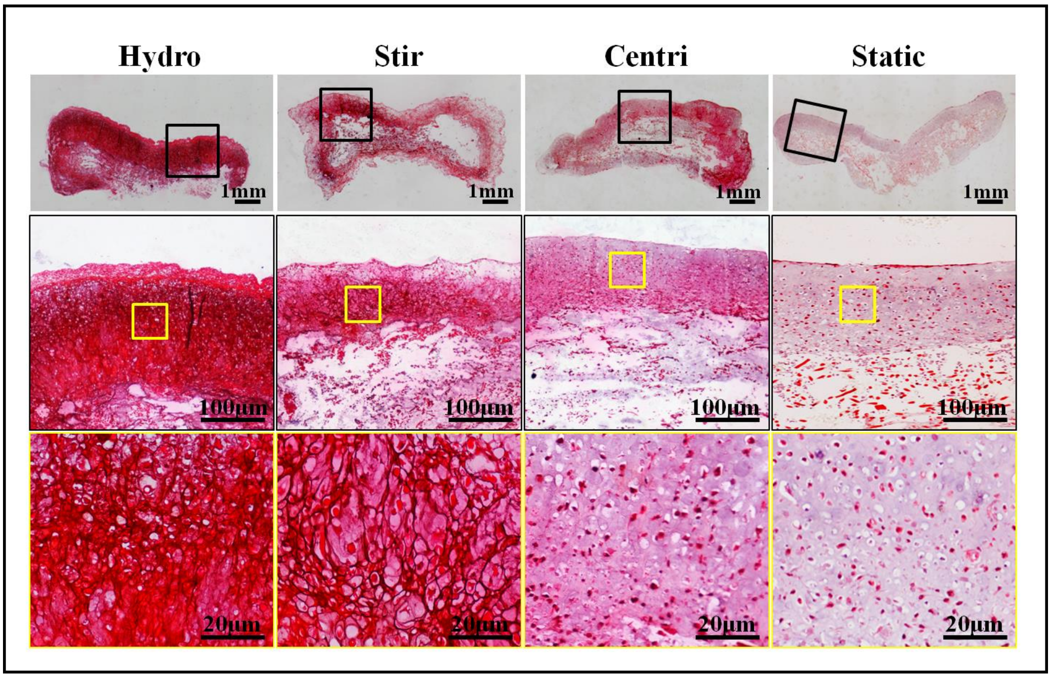

Figure S2: Safranin-O staining of vitro-EC. The sample in HP group shows strong positive staining of Safranin-O with relatively homogeneous cartilaginous structures. Positive staining of Safranin-O in Shear and Centri groups is observed mainly at the outer but not central regions. The sample in Static group only shows weak positive staining of Safranin-O at the outer region.

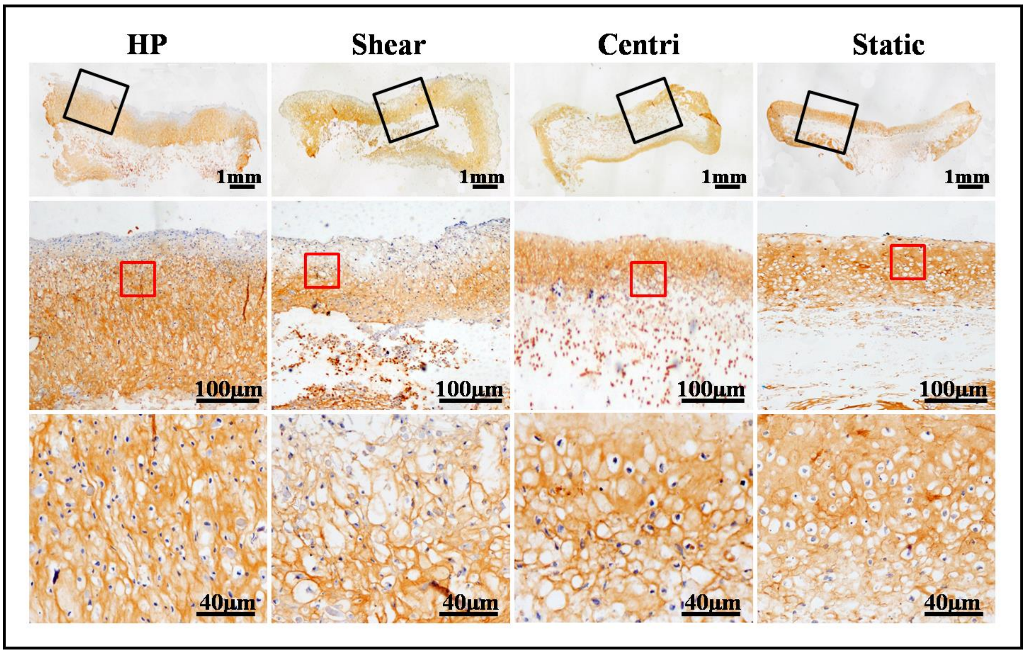

Figure S3: Collagen II staining of vitro-EC. The sample in HP group shows strong positive staining of collagen II with relatively homogeneous cartilaginous structures. Positive staining of collagen II in Shear and Centri groups is observed mainly at the outer but not central regions. The sample in Static group only shows weak positive staining of collagen II at the outer region.

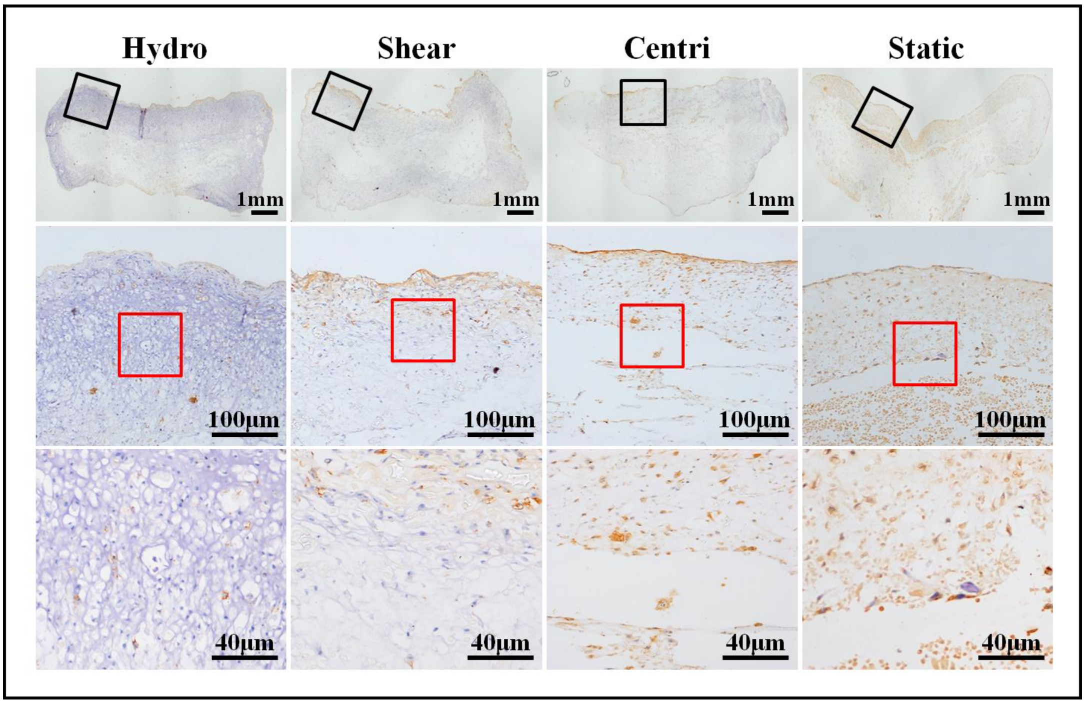

Figure S4: Collagen I staining of vitro-EC. The sample in HP group shows basically negative staining of type I collagen. Positive staining of collagen I in Shear and Centri groups is observed mainly at the outer regions of the samples. The sample in Static group shows strong positive staining of collagen I in the whole region.

Supplemental Table

Table S1. Primers for Real-Time RT-PCR

Gene	Forward Primer（5'-3')	Reverse Primer（5'-3')	Amplicon Size(bp)
**β-actin**	CAAGGAGAAGCTCTGCTACGTC	TGAAGGTGGTCTCGTGGATG	203
**LOX**	ACAGCATACAGGGCAGATGTCA	CTTGGTCGGCTTGGTAAGAAAT	107
